# A Neglected Disease: Hidradenitis Suppurativa a Rare Cause of Amyloidosis Complicated With Sepsis and Renal Failure: A Case Report

**DOI:** 10.1155/2024/4893040

**Published:** 2024-08-27

**Authors:** Orhun Demir, Omer Zuhtu Yondem, Mehmet Doganay

**Affiliations:** ^1^ Department of Anesthesiology Faculty of Medicine Lokman Hekim University, Ankara, Türkiye; ^2^ Department of Infectious Diseases Faculty of Medicine Lokman Hekim University, Ankara, Türkiye

## Abstract

**Background:** Hidradenitis suppurativa (HS) is a painful relapsing inflammatory disease characterized with subcutaneous nodules, abscesses, tunnels, and deforming scars. We present a case of HS complicated with amyloidosis who was admitted with sepsis and acute renal failure.

**Case Report:** A 53-year-old male patient with a history of HS and amyloidosis was accepted to the intensive care unit suffering from acute kidney failure and sepsis symptoms. He was receiving adalimumab during admission. He received hemodialysis and piperacillin–tazobactam antibiotherapy. After 15 days of hospitalization, his sepsis was treated, but he was taken to a routine hemodialysis programme.

**Conclusion:** In the presence of amyloidosis with intervenient infections, the risk of chronic renal failure in HS cases can be kept in mind even if the patient is receiving TNF inhibitors.

## 1. Background

Hidradenitis suppurativa (HS) is a painful relapsing inflammatory disease characterized with subcutaneous nodules, abscesses, tunnels, and deforming scars. It is generally seen after puberty. The global prevalence of HS varies from less than 1%–4% to 0.05% in the United States and 0.77–1.19 in the United Kingdom [1, 2]. AA amyloidosis associated with HS cases is relatively rare as well. Renal involvement in HS resulting from AA amyloidosis was first described in 1966 [3]. We present a case of HS complicated with amyloidosis who was admitted with sepsis and acute renal failure.

## 2. Case Report

A 53-year-old male patient with a history of atherosclerotic coronary arterial disease, chronic cardiac insufficiency, chronic HS, and heavy smoking for 35 years was admitted to the emergency department with altered mental status and elevated serum creatinine levels. The patient was accepted to the intensive care unit (ICU) with the clinical diagnosis of sepsis and renal failure. From his history, it was confirmed that HS was first diagnosed in 1995, and he underwent surgery several times due to lesions of HS at the gluteal region between 1995 and 1997. He underwent a gingival biopsy and was diagnosed with amyloidosis 1 month ago in another institution, and he has been receiving adalimumab until that time. He declared that adalimumab therapy did not result in a significant improvement in HS lesions when compared to previous therapies he received. His body mass index was 27.

**Figure 1:**
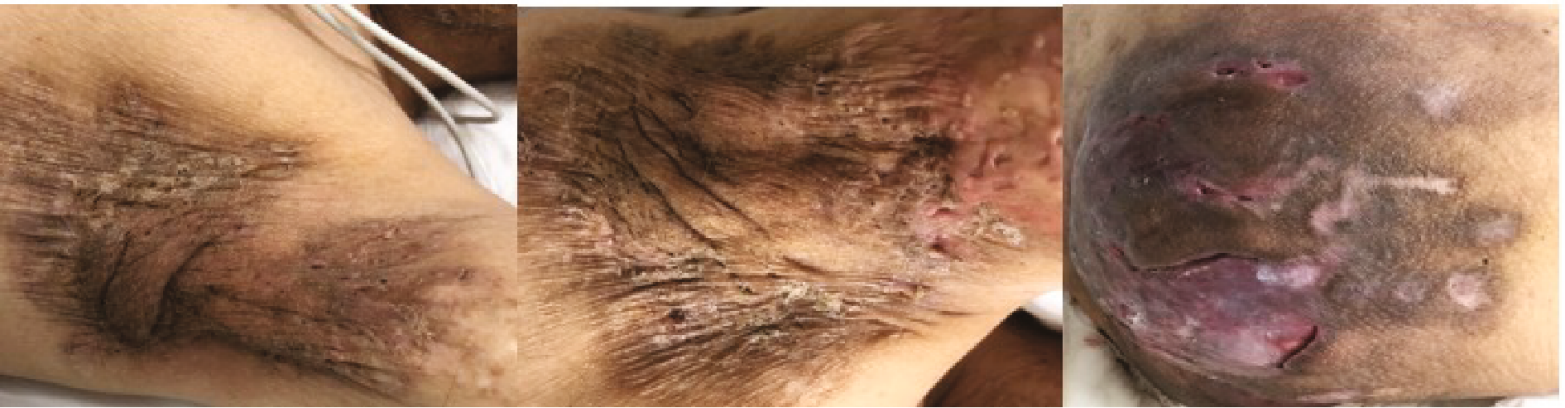
Purulent HS lesions with interconnected sinus tracts in both axillary and right gluteal regions.

He was in a toxic and pale appearance. He was confused with a Glasgow coma scale (GCS) of 11. He needed noradrenaline infusion for hypotension which was unresponsive to intravenous fluid administration. He was tachycardic and his saturation of O^2^ was slightly decreased to 94% with a need of 4 L per min O^2^ insufflation. Body temperature was 38.5°C. In his physical examination, there were signs of severe HS lesions (Hurley Stage III) which were purulent with interconnected sinus tracts in both axillary regions and the right gluteal region involving the rectum and waist (Figure 1). Culture samples from purulent lesions were sent for microbiological assessment. Some of his initial serum biochemical parameters were as follows: white blood cell (WBC) count: 15.01; serum creatinine: 11.32 mg/dL; urea: 182.2 mg/dL; blood urine nitrogen: 85.14 mg/dL; C-reactive protein (CRP): 176.4 mg/L; serum amyloid: 3.08 mg/dL; and procalcitonin in the normal range. His chest radiogram revealed pneumonia bilaterally. Ceftriaxone, teicoplanin, and clindamycin were started empirically and shifted to piperacillin and tazobactam according to culture results of lesions revealing *Escherichia coli* resistant to initial antibiotics on the fifth day. He received hemodialysis 5 times in his 12-day-long ICU stay. The patient refused the operation due to interfascial abscesses in the gluteal region because of the bad experience about the results of previous several surgical treatments. Finally, he was discharged to the nephrology department with a GCS of 13, WBC count in the normal range, reduced serum CRP levels to 20.37 mg/L, and without need for inotropic support. Unfortunately, renal impairment did not improve. We thought that the renal failure resulted from sepsis and underlying amyloidosis, but we could not detect the clear reason since we did not perform a renal biopsy. After 3 days of hospitalization, he was discharged from the hospital and taken to a routine hemodialysis programme.

## 3. Discussion

Although the global prevalence of HS is low, with its severe course and systemic comorbidities, HS affects the comfort and quality of patients' lives. Even increased suicide prevalence is reported in HS cases when compared to the normal population [4]. HS patients can experience some psychological problems such as social stigma, poor mental status, substance use disorder, and relation issues [5]. A low GCS and confusion were observed in admission in this case, but it was interpreted as a result of sepsis because his GCS dramatically increased after antibiotic treatment during discharge from the ICU.

Lesions of HS are seen mostly in axillary anogenital and inguinal regions [6]. In concordance with the literature, the patient had lesions in the axillary and anorectal regions. Hurley classification that determines the severity of the HS is shown in Table 1 [7]. According to Hurley's classification, the lesions were Stage III which reflects severe disease in our case. It has been reported that kidney disease incidence is higher in severe (3.1%) than in mild (1.5%) cases [8]. Proinflammatory cytokines such as interleukin (IL)-1, IL10, IL-17, and tumor necrosis factor (TNF)-*α* play important roles in HS physiopathology [5, 9]. Three clinical subtypes of HS have been reported: axillary-mammary, follicular, and gluteal. But this typification was not found to be useful for prognosticating treatment outcomes even though some recent literature defines a correlation between a higher risk for disease aggressiveness and follicular subtype and an inflammatory phenotype [5]. Nonspecific inflammatory parameters such as erythrocyte sedimentation, CRP, and leukocytosis-associating neutrophilia may reflect a systemic inflammatory burden for HS. The value of some new biomarkers such as Chitinase-3-like Protein 1 and S100A8/A9 to show the severity of the disease is controversial [5]. In our case, there was increased CRP and leukocytosis which could be related to hyperinflammation as a result of HS and coexisting sepsis.

Underlying diabetes mellitus, hypertension, obesity, previous cardiovascular disease, female gender, and history of smoking are determined as risk factors for chronic renal failure in HS patients [10, 11]. The patient had a history of smoking and previous cardiovascular disease in our case. Possible pathogenesis for chronic renal failure in HS cases is as follows: effect of Type 1 and Type 17 lymphocytes on tubular and mesangial cells of the kidney; impairment of basement membrane zone in kidneys resulting in glomerular hyperfiltration and proteinuria; and glomerulonephritis as a consequence of secondary renal amyloidosis [10]. Proteinuria could not be detected due to lack of urine in our case, but amyloidosis was diagnosed as a probable risk factor for chronic renal failure.

HS is associated with many systemic complications including anemia, fistulae to adjacent tissue, lymphoedema, squamous cell carcinoma, hypercalcemia, and secondary amyloidosis [9]. Secondary systemic amyloidosis is characterized by the accumulation of Amyloid A which is a nonimmunoglobulin protein synthesized in the liver. Amyloidosis most commonly affects the kidneys, but the spleen, adrenal glands, liver, heart, and joints can be involved as well [9]. The prevalence of amyloidosis was found 11-fold higher (0.2%) when compared with the normal population in HS cases. Amyloidosis is generally manifested as nephrotic syndrome or proteinuria [9]. The incidence of renal insufficiency in HS patients with amyloidosis is reported as 44.4% (which seems very high), and TNF inhibitors particularly adalimumab were found to have favorable outcomes in avoiding renal failure in a review article [6]. In our case, the use of adalimumab could not prevent renal insufficiency probably as a result of coexisting sepsis.

## 4. Conclusion

HS is a rare disease and can result in different systemic comorbidities if not well treated and followed up. In the presence of amyloidosis with intervenient infections, the risk of chronic renal failure can be kept in mind even if the patient is receiving TNF inhibitors.

## Figures and Tables

**Table 1:** The Hurley staging system and the physician global assessment for hidradenitis suppurativa scale.

Hurley Stage
Stage I: Solitary or multiple isolated abscess formation without scarring and any sinus tract formation
Stage II: Recurrent abscess, single or multiple widely separated lesions with the formation of sinus tracts and cicatrization
Stage III: Diffuse and broad involvement across a regional area with multiple interconnected sinus tracts and abscess
